# *Borrelia* spirochetes in European exotic farm animals

**DOI:** 10.3389/fvets.2022.996015

**Published:** 2022-09-28

**Authors:** Johana Hrnková, Marina Golovchenko, Abubakar Sadiq Musa, Tersia Needham, Jignesh Italiya, Francisco Ceacero, Radim Kotrba, Libor Grubhoffer, Natalie Rudenko, Jirí Cerný

**Affiliations:** ^1^Faculty of Tropical AgriSciences, Czech University of Life Sciences Prague, Prague, Czechia; ^2^Institute of Parasitology, Biology Center, Czech Academy of Sciences, Ceské Budějovice, Czechia; ^3^Department of Ethology, Institute of Animal Science, Prague, Czechia; ^4^Faculty of Sciences, University of South Bohemia, Ceské Budějovice, Czechia

**Keywords:** tick, tick-borne pathogens, *Ixodes ricinus*, tick hosts, *Borrelia burgdorferi* sensu lato, *Borrelia miyamotoi*

## Abstract

Ticks transmit a broad spectrum of pathogens, threatening both animal and human health. Tick survival and proliferation are strongly dependent on host selection and suitability. The hard tick *Ixodes ricinus*, which is widespread throughout most of Europe, is a host generalist capable of feeding on many different vertebrate species. Pasture-kept exotic farm animals may be at a high risk for tick and tick-borne pathogens infestations but research characterizing this is currently lacking. This study focused on the detection of *Borrelia* spirochetes (including *Borrelia miyamotoi*) in exotic farm animals. Using nested-PCR with *Borrelia*-specific primers, 121 serum samples from 54 exotic farm animals of several species bred in four different farms in Bohemia and Moravia (Czechia) were tested. Positive samples were sequenced for the identification of *Borrelia* species. The prevalence of *Borrelia* DNA in the samples ranged from 13 to 67%, depending on the sampling site. The sequencing results confirmed the DNA presence of multiple spirochete species from the *Borrelia burgdorferi* sensu lato complex. Only one sample from an ostrich (*Struthio camelus*) was found to be positive for *Borrelia myiamotoi*. The results show that exotic farm animals can serve as hosts for hard ticks and can be infected by *Borrelia* spirochetes, transmitted by hard ticks. Therefore, these animals could play a relevant role in maintaining *Borrelia* spirochetes in nature.

## Introduction

Ticks are important hematophagous ectoparasites and vectors of several pathogens affecting both animal and human health. The presence of suitable vertebrate hosts is one of the driving forces determining tick survival in a region and subsequent circulation of tick-borne pathogens (1). From the perspective of host-preference, ticks can be characterized as either host generalists or specialists (2). Most tick species of veterinary and medical interest are host generalists, with the potential for transmitting tick-borne pathogens (TBPs) to a wide range of hosts. Availability and diversity of suitable hosts is closely linked to tick prevalence, so generalists have the “upper hand” in establishing stable populations in much larger areas compared to highly specialized tick species (1). The correct assessment and knowledge of tick-host interactions and TBP prevalence in host populations is vital for understanding the ecoepidemiology of tick-borne diseases and the population dynamics of ticks.

The present research study was conducted in Czechia, where the hard tick *Ixodes ricinus* is the predominant tick species. *I. ricinus* is widespread across Europe and is acknowledged as a typical generalist capable of feeding on as many as 300 different vertebrate species (3, 4). The selection of hosts by ticks, however, is not completely random (5). The factors influencing host selection are numerous and include: tick development stage, size, the geographical range of host and its health state, season, temperature and even infections of the tick with selected pathogens that can modulate tick behavior (6–9). The formation of host-specific races has also been confirmed in some *I. ricinus* populations (7). The tick-host relationship of *I. ricinus* is rather complex and new influencing factors are continually being discovered.

Apart from the primary hosts of *I. ricinus*, like red deer (*Cervus elaphus*), wild boars (*Sus scrofa*), yellow-necked mice (*Apodemus flavicollis*), European hares (*Lepus europaeus*), voles, some incidental host species have been studied in recent years. Animals originating outside of the natural *I. ricinus* geographical range (i.e., animals that are non-indigenous or exotic to Europe, typically kept in zoos) are also prone to *I. ricinus* infestation and can harbor some of the common TBPs within the region (10–13). These findings suggest that, due to the high adaptability of *I. ricinus* to different hosts, all animals kept within the endemic zone of *I. ricinus* (even house pets or accompanying animals) should be considered as potential sources of TBPs. It is still unclear as to whether exotic animals are competent reservoirs and can serve as a source of infection for uninfected tick vectors. Ticha et al. (13) confirmed the sensitivity or resistance of tested *Borrelia* spirochetes to serum complements of several zoo animals, suggesting a tolerance to *Borrelia* infection in at least some exotic species and a potential reservoir competence of these animals. Studies conducted on animals that are exotic to Europe have thus far only focused on zoological gardens, and to the best of our knowledge, no studies concerning exotic species kept on private farms have been published to date. Exotic animals are farmed for a variety of reasons, including production, for example milk (buffalo species), fiber (llama species), or meat (ostrich, buffalo, antelope species). Hobby farms and private zoo parks are also common in Czechia, maintaining animals like camels, llamas, donkeys, ostriches, and others (Table 1). These private establishments provide much closer animal-human-nature contact compared to zoological gardens. Furthermore, these establishments are often located in rural or even forested landscapes, unlike zoological gardens which are more often located in urban or peri-urban areas. These localities make exotic animal farms and private zoos ideal hotspots for tick-exotic host interactions, providing an increased likelihood of TBP circulation.

**Table 1:** The results of *Borrelia* testing and tick collections on selected exotic animal farms in Czechia.

**Farm**	**Ticks on pasture**	**Ticks around the** **farm**	**Prevalence of** ***Borrelia* in** **animals**	**Animal (ID)**	Age^*^	**Sex**	**Positive/** **Negative**	***Borrelia* species** **found**	**Overall prevalence** **on farm**
Hobby camel farm, south Bohemia	1 nymph	11 (5 females, 5 males, 1 nymph)	Llamas; tested: 4, positive: 2, 50%	Llama 1	115 months	F	Positive	*Borrelia burgdorferi* sensu stricto (B. ss)	38%
				Llama 2	127 months	M	Positive	*Borrelia garinii*	
				Llama 3	19 months	F	Negative	/	
				Llama 4	19 months	F	Negative	/	
			Bactrian camels; Tested: 10, Positive: 3, 30%	Bactrian camel 1	91 months	F	Positive	*B. ss*	
				Bactrian camel 2	127 months	F	Positive	*B. ss*	
				Bactrian camel 3	79 months	F	Positive	*Borrelia afzelii*	
				Bactrian camel 4	139 months	M	Negative	/	
				Bactrian camel 5	115 months	F	Negative	/	
				Bactrian camel 6	175 months	M	Negative	/	
				Bactrian camel 7	55 months	F	Negative	/	
				Bactrian camel 8	79 months	M	Negative	/	
				Bactrian camel 9	67 months	F	Negative	/	
				Bactrian camel 10	283 months	F	Negative	/	
			Dromedary camel; Tested: 1, Positive 1, 100%	Dromedary camel	55 months	M	Positive	*B. ss*	
Experimental antelope farm, central Bohemia	1 nymph	0	Elands; Tested: 25, Positive: 11, 44%	Eland antelope 249	17 months	F	Positive	*B. ss*	44%
				Eland antelope 251	17 months	M	Positive	*B. ss*	
				Eland antelope 253	17 months	F	Positive	*B. afzelii*	
				Eland antelope 255	17 months	F	Positive	*B. garinii + B. bisetti*	
				Eland antelope 258	16 months	M	Positive	*B. ss + B. garinii + B. bisetti*	
				Eland antelope 259	16 months	M	Positive	*Borrelia americana*	
				Eland antelope 261	16 months	F	Positive	*B. afzelii*	
				Eland antelope 267	17 months	M	Positive	*B. ss*	
				Eland antelope 268	17 months	F	Positive	*B. afzelii*	
				Eland antelope 269	16 months	M	Positive	*B. afzelii*	
				Eland antelope 272	15 months	M	Positive	*B. ss + B. bisetti*	
				Eland antelope 248	17 months	F	Negative	*/*	
				Eland antelope 250	17 months	F	Negative	*/*	
				Eland antelope 252	17 months	F	Negative	*/*	
				Eland antelope 254	17 months	M	Negative	*/*	
				Eland antelope 257	16 months	M	Negative	*/*	
				Eland antelope 260	16 months	M	Negative	*/*	
				Eland antelope 262	16 months	F	Negative	*/*	
				Eland antelope 266	8 months	M	Negative	*/*	
				Eland antelope 271	3 months	M	Negative	*/*	
				Eland antelope 273	2 months	F	Negative	*/*	
				Eland antelope 231	20 months	F	Negative	*/*	
				Eland antelope 207	32 months	F	Negative	*/*	
				Eland antelope 219	29 months	F	Negative	*/*	
				Eland antelope A	19 months	F	Negative	*/*	
Milk and meat Buffalo farm, central Bohemia	2 males + 1 female feeding on animal	11 (3 females, 6 males, 2 nymph)	Buffaloes; Tested: 8, Positive: 1, 13%	Buffalo 19	43 months	F	Negative	*/*	13%
				Buffalo 3	115 months	M	Negative	*/*	
				Buffalo 4	91 months	F	Negative	*/*	
				Buffalo 11	67 months	F	Negative	*/*	
				Buffalo 21	31 months	F	Negative	*/*	
				Buffalo 13	67 months	F	Negative	*/*	
				Buffalo 14	67 months	F	Negative	*/*	
				Buffalo 18	55 months	F	Positive	*B. burgdorferi* sensu lato	
Meat ostrich farm, Moravia	N/A	N/A	Ostriches; Tested: 6, Positive: 4, 67%	Ostrich 1	14 months	M	Positive	*B. garinii*	67%
				Ostrich 2	16 months	F	Positive	*B. garinii*	
				Ostrich 3	16 months	M	Positive	*B. burgdorferi* sensu lato	
				Ostrich 8	13 months	M	Negative	*/*	
				Ostrich 6	16 months	M	Negative	*/*	
				Ostrich 4	12 months	M	Positive	*Borrelia miyamotoi*	

* in 2020.

## Materials and methods

### Serum sample collection

A total of 121 blood samples were collected from 54 exotic farm animals of different species (Table 1). Vacutainers (8 ml CAT Serum Sep Clot Activator, Vacuette, Greiner Bio-One GmbH, Austria) were used for serum collection. The blood samples from live animals were obtained during regular annual health status inspection by external veterinarians according to the recommendations of the State veterinary authority of the Czech Republic. Ostrich samples were obtained after slaughter of the animals. Moreover, samples from captive-bred common eland (*Taurotragus oryx*) were collected at the Czech University of Life Sciences Research farm at Lány, Central Bohemia, in the Czech Republic which is accredited for experiments on animals (Permit: 63479_2016-MZE-17214). Specific protocol of approval was obtained by the Institutional Animal Care and Use Committee of the Czech University of Life Sciences, Prague (clearance no. CZU 20/19). The institutions and animal owners agreed to participate in this study and provided written informed consent. After blood withdrawal, the samples were delivered to the laboratory, and serum was separated by centrifugation (10 min at 1,000 rpm) at room temperature. The serum was then refrigerated at 4°C (if used the next day) or frozen at −20°C until analysis.

The animals sampled included: 25 common elands (*Taurotragus oryx*), 6 common ostriches (*Struthio camelus*), 8 water buffaloes (*Bubalus bubalis*), 1 dromedary camel (*Camelus dromedarius*), 10 Bactrian camels (*Camelus bactrianus*), and 4 llamas (*Lama glama*) (Table 1). The location and husbandry practices of the selected exotic animal farms were variable. The Common Eland Research Facilities (CERF) of the Czech University of Life Sciences Prague (CZU) and the buffalo milk and meat farm are both located in Central Bohemia, and had similar husbandry conditions, with short grass grazing in outdoor paddocks and the provision of supplementary feeding of a complete ration in adjacent stables. The buffalo farm is located on the edge of a forested area, while the eland farm is surrounded by agricultural fields and an asphalt road. The hobby camel farm in Southern Bohemia, and the ostrich meat farm in Moravia, had more extensive outdoor paddocks with both short and long grass cover, and supplementary feed provided in their stables. The camel farm was also situated close to the edge of a forest. The animals were free to roam in and out of their stables during most of the year, except for aggressive males or sick animals which had their own smaller paddocks.

Most collections were conducted once per farm during the active *I. ricinus* season at the time of scheduled routine veterinary health inspections. Eland blood sampling was conducted over 3 consecutive months at the CZU Common Eland Research Facilities during summer, from July to September 2020 (78 samples in total from 25 individual animals). All sampled elands were juveniles (3–17 months old) born on the farm.

### Live ticks collection

Live ticks were collected on each farm using the flagging method (14) on both the pasture areas and the areas immediately surrounding the farms (Table 1). Only *I. ricinus* ticks were collected; no other tick species were identified during the collections. Flagging was conducted in unified timeframes of 1 h per pasture and 1 h per surrounding area. The collected live ticks were sorted according to their developmental stage and sex, and then stored live at 4°C for further testing. Live tick collection directly from the live animals was mostly impossible due to the handling methods of the animals (crushes with solid sides). However, when possible, live ticks were collected.

### Nested polymerase chain reaction (nested-PCR)

Ticks were homogenized using a Tissue Lyser (Qiagen). DNA extraction from the biological samples was performed using a DNeasy Blood & Tissue Kit (Qiagen) according to the manufacturer's protocol.

All samples were screened using PCR for the presence of DNA from the *Borrelia burgdorferi* s.l. complex, including the species *Borrelia burgdorferi* sensu stricto, *Borrelia garinii, Borrelia afzelii, Borrelia americana*, and *Borrelia bisetti*. The DNA presence of the relapsing fever spirochete *Borrelia miyamotoi* was also tested. The presence of *Borrelia burgdorferi* s.l. was examined by PCR amplification of partial *ospC* and *flagellin* genes using gene-specific primers. In the case of low-quality sequences, some samples were further re-examined through the amplification of partial *p66* gene. All PCR reactions were conducted in two steps (nested PCR).

Whole EDTA blood could have been used for *Borrelia* detection, but the results would have been unreliable. Since spirochetes causing Lyme borreliosis are not intracellular pathogens, the presence of inhibitory non-target DNA in blood cells would bias the PCR test results toward false negatives. Removal of red blood cells resulted in a reduction of the amount of non-target DNA in the sample, yielding more accurate results. The reliability of the protocol chosen has been shown in numerous previous studies (13, 15–17) and is optimized for utilizing animal and human serum, resulting in an increased sensitivity of detection for LB spirochetes. Since serum was easily obtainable through routine veterinary examination of the animals and was one of the least invasive but still clinically valid sample types, it was evaluated as the best available option for the detection of *Borrelia* DNA in these animals.

The fragment of the *ospC* gene was amplified by spacer/nested PCR using previously described primers and reaction conditions (18). The first round was conducted using primers targeting a 617 bp long fragment (Table 2); the conditions of both reactions were the same (except the annealing temperature, which was 50 and 52 °C for the first and the second round of PCR, respectively): 30 cycles at 95 °C for 30 s, 50/52 °C for 30 s, and 72 °C for 30 s.

**Table 2:** Selected primers used for *Borrelia* detection.

**Target gene**	**Specification**	**Primer sequence**	**Source**
Flagellin gene	Out Fw	5′-GCATCACTTTCAGGGTCTCA-3′	(19)
Flagellin gene	Out Rv	5′-TGGGGAACTTGATTAGCCTG-3′	(19)
Flagellin gene	In Fw	5′-CTTTAAGAGTTCATGTTGGAG-3′	(19)
Flagellin gene	In Rv	5′-TCATTGCCATTGCAGATTGT-3′	(19)
ospC gene	Out Fw	5′-ATGAAAAAGAATACATTAAGTGC-3′	(18)
ospC gene	Out Rv	5′-ATTAATCTTATAATATTGATTTTAATTAAGG-3′	(18)
ospC gene	In Fw	5′-TATTAATGACTTTATTTTTATTTATATCT-3′	(18)
ospC gene	In Rv	5′-TTGATTTTAATTAAGGTTTTTTTGG-3′	(18)
p66	Out Fw	5′-GATTTTTCTATATTTGGACACAT-3′	(18)
p66	Out Rv	5′-TGTAAATCTTATTAGTTTTTCAAG-3′	(18)
p66	In Fw	5′-CAAAAAAGAAACACCCTCAGATCC-3′	(18)
p66	In Rv	5′-CCTGTTTTTAAATAAATTTTTGTAGCATC-3′	(18)
glpQ^*^	Out Fw	5′-ATGGGTTCAAACAAAAAGTCACC-3′	(20)
glpQ^*^	Out Rv	5′-CCAGGGTCCAATTCCATCAGAATATT-3′	(20)
glpQ^*^	In Fw	5′-ATGGGTTCAAACAAAAAGTCACC-3′	(20)
glpQ^*^	In Rv	5′-GATGTCTTTACCTTGTTGTTTATGCCA-3′	(20)

* Targeting Borrelia miyamotoi.

Primers described by Wills et al. (19) were used to amplify a 447 bp long fragment of the *flagellin* gene (Table 2). The cycling conditions of both reactions were the same (except the annealing temperature that was 55 and 58 °C for the first and the second round of PCR, respectively): 30 cycles at 95 °C for 30 s, 55/58 °C for 30 s, and 72 °C for 30 s.

A 684 bp long fragment of the *B. burgdorferi p66* gene was amplified by nested PCR using primers according to Bunikis et al. (18) (Table 2). PCR conditions were the same for both rounds of PCR: 95°C for 5 min followed by 30 cycles of 95°C for 30 s; 50°C for 30 s; and 72°C for 1 min.

The presence of *B. miyamotoi* was also tested by nested PCR for the glycerophosphodiester phosphodiesterase (*glpQ*) *B. miyamotoi* gene, using the protocol described earlier (20) and producing 480 or 461 bp long fragments (Table 2) under the following amplification conditions: 30 cycles at 95 °C for 30 s, 52 °C for 30 s, and 72 °C for 30 s for the first round of PCR, and 25 cycles at 95 °C for 30 s, 55 °C for 30 s, and 72 °C for 30 s for the second round of PCR.

In all cases, a reaction mix without a DNA template was used as a negative control, and purified DNA from *Borrelia* cultures was used as a positive control, containing a mix of *B. burgdorferi* s.l. complex species. A positive control for *B. miyamotoi* was obtained by the isolation of DNA from infected ticks or by using the cloned *glpQ* gene in a vector plasmid. The PCR reactions were carried out in a final volume of 20 μl, using 2× HotStarTaqPlus Master Mix (Qiagen). Amplicons were visualized by electrophoresis on a 1.5 % agarose gel (1× TAE, pH 8.0) stained with either SYBR^®^ Gold DNA gel stain (Invitrogen) or EtBr (Ethidium bromide). DNA extraction steps, PCR, and post-amplification analyses were performed in separated areas of the laboratory with all necessary precautions taken against contamination.

Polymerase chain reaction products of the expected sizes were excised from agarose gels, purified using Centrifugal Filter Units (ULTRAFREE DNA Extraction from Agarose, Millipore) and sequenced in both directions, using the same primers as those used for the PCR. Sequence analysis was performed by SeqMe (SEQme s.r.o., Czech Republic) and the sequences were compared to those available in the GenBank^™^ dataset by Basic Local Alignment Tool (BLAST) analysis.

## Results

The prevalence of *Borrelia* spp. infection was different according to the farm. Of the 54 animals tested, 22 were found positive for one or more *Borrelia* species (Table 1). The overall prevalence of *Borrelia* infection was therefore 41%. *Borrelia burgdorferi* sensu stricto was most common and was found in 9 of the 22 positive animals. Among other detected *Borrelia* species were *Borrelia garinii, Borrelia afzelii, Borrelia bisetti, Borrelia americana, Borrelia burgdorferi* sensu lato, and *Borrelia miyamotoi* (Table 1). The collection times and sample sizes differ between each farm; therefore, the presented order of prevalence is chosen primarily to provide a better overview of the results and does not indicate susceptibility of a given animal species to *Borrelia* infections.

The highest prevalence 67%, with the 95% confidence interval (CI) from 47 to 86%, *Borrelia* prevalence, was reported for the ostrich samples, where 4 of the 6 ostriches sampled tested positive for *Borrelia* spp., including one which was positive for *B. miyamotoi*. The detection of *B. miyamotoi* in this ostrich was unique and no other sample in this study tested positive for *B. miyamotoi*.

The second-highest prevalence (44%, CI 34–54%) was observed in the common eland located in Central Bohemia. Of the 25 animals sampled, 11 were positive for the presence of DNA from the *B. burgdorferi* sensu lato spirochetes, including several co-infections with multiple *Borrelia* species (Table 1). Of the positive animals herein found, most were repeatedly positive (55%, CI 40–70%) during the entirety of the collection period (Table 3). Animals no. 259 and 267 were negative at the start of the monitoring period but were infected in September 2020. Animal no. 268 was negative from September 2020, possibly suppressing the preceding *Borrelia* proliferation. Animal no. 261 seemingly suppressed the infestation in August 2020, but a positive result was found again in September 2020, suggesting a relapse or novel reinfection (Table 3). The unusual result for animal no. 261 might have also been caused by the faulty isolation or degradation of DNA from the serum sample collected in August. A statistical analysis of the influence of *Borrelia* infection on the eland body condition scores and average daily weight gains during the study period showed no statistically significant effect on these parameters in connection to either ongoing or acquired infection. Only one tick nymph was collected from the eland paddocks, despite repeated flaggings during different periods of tick activity. No feeding ticks were observed on the animals, but the leg and abdominal regions were not thoroughly inspected due to the handling method used (restraint box/squeeze with solid sides).

**Table 3:** Results of *Borrelia* testing in the eland antelope during a 3-month sampling period.

**Animal ID**	**July 2020**	**August 2020**	**September 2020**
249	Positive	Positive	Positive
251	Negative	N/A^*^	Positive
253	Positive	Positive	Positive
255	Positive	Positive	Positive
258	Positive	Positive	Positive
259	Negative	Negative	Positive
261	Positive	Negative	Positive
267	Negative	Negative	Positive
268	Positive	Positive	Negative
269	Positive	Positive	Positive
272	Positive	Positive	Positive

* Animal could not be tested.

The third-highest prevalence (40%, CI 27–53%) was found at a camel hobby farm located in Southern Bohemia. Two llamas, three Bactrian camels, and one dromedary camel tested positive, from the fifteen animals tested. No clinical symptoms of borreliosis were observed in any of these animals and they were generally in good health. Live ticks were found in both the pasture (one) and surrounding areas (eleven), but none were found on the animals themselves (Table 1).

Finally, the lowest prevalence (13%, CI 1–24%) was observed at the buffalo milk and meat farm in Central Bohemia, where only one out of eight animals tested positive for *Borrelia*. Three ticks were found in the pasture, of which one was found feeding on a buffalo. Eleven ticks were found in the surrounding areas.

Two Generalized Linear Models with binary response tested the effect of age (in months), sex, and farm or region (one in each model) on the occurrence of *Borrelia* infection. None of the factors were significant, so these effects were disregarded.

## Discussion

The tick-host-pathogen network is a complicated, multifactorial biological system, as each component has specific characteristics that potentially influence one another. This complicates the research thereof and has resulted in many influencing factors being currently unknown. The relationships between ticks, pathogens, and their hosts have been studied intensively in household pets, livestock, wildlife, and humans (21–24). Animals kept in zoos in different countries and climates have also been studied, although less thoroughly (10–13). Farm-kept exotic animals have not been considered up until now, in this otherwise extensive research field. Exotic, non-indigenous animal farms can represent one more piece of the puzzle regarding the biology of ticks and TBPs, because they are introducing a new, atypical, and evolutionarily unadapted host into the European tick-host-pathogen network. Since this study has confirmed that such animals can carry some European *Borrelia* species, and can be infested with ticks, it is suggested that this topic be furtherly investigated.

In their natural habitats, the exotic animal species tested in the present study show varied susceptibility or immunity to tick bites or TBPs. In Africa, eland and other wildlife species developed alongside local tick species for thousands of years, providing them with increased immunity to tick infestations (25, 26). On the other hand, common eland show larger tick infestation numbers in comparison to other wild African large mammal species, possibly suggesting that this antelope is preferred by ticks as a potential host (26, 27). African wild ungulate species are often blamed for the increase of tick numbers in mixed livestock-wildlife farming enterprises; however, no clear evidence of correlation between the presence of wildlife species and tick numbers has been documented (26, 28). In Indian water buffaloes, close relatives of European milk buffaloes, the tick prevalence observed decreased with age and differed between the sexes, suggesting that adult females are less suitable hosts for ticks (29, 30). In dromedary camels (*Camelus dromedarius*), the highly specialized tick, *Hyalomma dromedarii*, is of the biggest concern in the Middle East (31); however, these camels show susceptibility to other tick species as well (32). Since dromedaries are extinct in the wild and are bred solely in captivity, it is difficult to assess their overall resistance to ticks and TBPs, since they inhabit such diverse climates and encounter different tick species. In Bactrian camels (*Camelus bactrianus*), even less information is available about their susceptibility to ticks. Several studies show that these camels harbor some of the well-known TBPs, including *Borrelia* spp (33, 34). Llamas (*Lama glama*) were experimentally confirmed to have the ability to host *Boophilus microplus* ticks (35), and tick paralysis was observed on llamas in the United States and Australia (36); however, there is limited knowledge about their tick resistance or susceptibility in the wild populations.

The lack of interest in farm-kept exotic animals in Europe is surprising for several reasons. First and foremost, animals kept on such establishments are often bred for meat and/or milk production for human consumption. This might pose a threat to human health if no appropriate tick and TBPs prevention strategies are implemented. It is known that tick-borne encephalitis virus (TBEV) is one of the possible alimentary infections from the wide variety of tick-borne infections. On several occasions, TBEV has been transmitted in unpasteurized milk and cheese from goats, cows, and other farm animals (37–40). This specific example might pose a risk for food-borne transmission, especially on smaller, extensive buffalo farms, where the animals are often kept in enclosures without any tick control and in proximity of ideal tick habitats. In this study, a tick was found feeding on a milk-producing buffalo, and a positive case of *Borrelia* infection was also confirmed in one of the animals; thus, it is safe to assume that these animals come into direct contact with the vectors of TBEV and theoretically could serve as potential infection sources. However, there are still many important gaps in knowledge about buffalo susceptibility and reservoir competence for TBPs, and thus more research needs to be conducted to support this hypothesis.

Secondly, exotic farm-kept animals could participate in the tick-host-pathogen system to some extent. Creation of natural foci of various TBPs in the proximity of exotic animal farms is possible, when we consider the combination of a constant abundance of hosts and good ecological conditions that provide the ideal environment for tick breeding (41, 42). Since the present study focuses solely on the prevalence of *Borrelia* in the selected animals, it is impossible to assess if all TBPs would show the same pattern of prevalence in exotic farm animals, which is one of the limiting factors of this research. Moreover, the reservoir competence, probably the most important factor of TBP host research, remains to be confirmed in these species as well. Efficient reservoir hosts can be characterized by many factors: they are abundant in tick-preferred habitats and can host many vector ticks; pathogens can survive and multiply in the reservoir host for a prolonged period of time or even for a lifetime, and they do not develop a resistance to tick bites even after repeated feedings (13, 43, 44). Since small-scale farms are often located in tick-suitable areas, the possibility of exotic animal species being suitable reservoir hosts is heightened, and should be examined further.

Finally, the current results from the eland research facilities presented another controversial topic. The eland facility is located in an area where the conditions for tick survival were sub-optimal, and only one tick was collected at the location despite repeated attempts during the study period. These results, combined with the high prevalence of *Borrelia* spp., raise an important question about the origin of the infection. Several answers could be offered regarding this discrepancy: one is the hypothesis of congenital or sexual transmission of the pathogen. In the case of *Borrelia*, this mode of transmission has been suggested due to the close evolutionary relationship with *Treponema pallidum*, a bacterium causing syphilis in humans (45, 46). The biological profile similarities of the two bacteria still raise suggestions that the process of congenital or sexual transmission, closely paired with teratogenicity, could be similar (47) even though this has largely been disproven in more recent research (48–50). A great deal of controversy surrounds this topic, as several studies confirm the congenital or sexual transmission of *borrelia* in both humans and animals (51–55), but some studies could not find any supporting evidence for the vertical transmission of *borrelia*, even in controlled laboratory animal studies (56, 57). The obvious absence of live ticks on the farm contrasting with the high prevalence of *Borrelia* infections could suggest the susceptibility of the eland to vertical transmission of the pathogen. This hypothesis is further supported by the fact that all animals sampled were young calves, 3–17 months old, born and raised on the farm, and thus possible contact with tick vectors is reduced compared to the adults as some of the parents had different places of birth and were imported from other facilities, where they could have had contact with infected ticks. Transmission of *Borrelia* through nursing is improbable, as some human studies suggest (51, 58). Another feasible explanation of this phenomenon might be a bit more straightforward, as many synanthropic animal species like feral cats (*Felis catus*) can access the facilities. The local feral cat population could be infested with ticks, since no anti-acaricidal treatment is usually provided, which could lead to the occasional introduction of non-attached ticks from these feral cats to the eland.

This brief research has shown that exotic farm animals in Europe are susceptible to *I. ricinus* and *Borrelia* spirochetes infection. Our findings suggest that these animals might play a role in the tick-host-pathogen network. We hope that the obtained results will prompt further research focusing on other important TBPs and exotic animal species that were not involved in this research.

## Data availability statement

The original contributions presented in the study are included in the article/supplementary material, further inquiries can be directed to the corresponding author/s.

## Ethics statement

The animal study was reviewed and approved by Czech University of Life Science Animal Welfare and Clearance Committee, Kamýcká 129, 165 00 Praha 6-Suchdol. Written informed consent for participation was not obtained from the owners because informed consent was obtained verbally when the animal owners were consulted about the research. All animal handling (blood withdrawal) was conducted during routine veterinary inspections by veterinarians known by the animal owners.

## Author contributions

JH, JC, and LG: research conceptualization and design. JH, JC, MG, AM, FC, JI, TN, and RK: data collection. JH, NR, JC, FC, MG, and RK: analysis and interpretation of results. JH: manuscript draft preparation. FC: statistical analysis. JC, NR, and LG: supervision. TN: language corrections. All authors contributed to the article and approved the submitted version.

## Funding

This work was supported by the Internal Grant Agency (IGA) FTZ CZU, Prague (IGA-20223107 and 20223108).

## Conflict of interest

The authors declare that the research was conducted in the absence of any commercial or financial relationships that could be construed as a potential conflict of interest.

## Publisher's note

All claims expressed in this article are solely those of the authors and do not necessarily represent those of their affiliated organizations, or those of the publisher, the editors and the reviewers. Any product that may be evaluated in this article, or claim that may be made by its manufacturer, is not guaranteed or endorsed by the publisher.
